# Heart transplantation management in northern Italy during COVID‐19 pandemic: single‐centre experience

**DOI:** 10.1002/ehf2.12874

**Published:** 2020-07-10

**Authors:** Alessandro Fiocco, Matteo Ponzoni, Raphael Caraffa, Massimiliano Carrozzini, Lorenzo Bagozzi, Matteo Nadali, Olimpia Bifulco, Giuseppe Toscano, Angela Pompea Fraiese, Tomaso Bottio, Gino Gerosa

**Affiliations:** ^1^ Cardiac Surgery Unit, Department of Cardiac, Thoracic, Vascular Sciences and Public Health University of Padua Padova Italy

## Introduction

Northern Italy is currently on the front line of European COVID‐19 outbreak and the first western region whose solid organ transplant programs have had to face the virus pandemic emergency.^1^

Data on heart transplanted patients' management in the COVID‐19 era are still very limited. Therefore, the ability to grant best clinical practice in transplant population should answer to crucial questions, mainly regarding immunosuppression‐related issues.^2^

First, severe infections in the immunocompromised population dramatically influence patient's prognosis. There is concern for the great variability which dominates the clinical presentation patterns in this population and the risk factors for severe infection have not been fully characterized.

Italian Transplant Authority (Centro Nazionale Trapianti, CNT) released guidelines on donor management suggesting real‐time reverse‐transcription polymerase chain reaction (rRT‐PCR) assays on nasopharyngeal swab (NPS) or bronchoalveolar lavage 24–48 h before organs retrieval, to identify and exclude SARS‐CoV‐2 positive donors.^3^ Conversely, no specific indications for recipients have been released so far.^4^

Although mortality data are not yet available in transplant recipients, outcome could be worse than in immunocompetent patients. For this reason, given for granted the negative status of the donor, our efforts should focus on assuring negativity of the recipient.

In this document, we summarize our adopted strategies to mitigate the impact of the current pandemic based on our single‐centre experience.

Particularly, given CNT ensured indication about donor management, we will focus on the recipient's list management and early postoperative treatment.

## Waiting list recipients' management protocol

### Epidemiological risk factors assessment and prevention

- Limit to one single caregiver for each patient. Interpersonal contacts should be limited.
- Daily home monitoring of temperature and symptoms should be performed from the patient or the caregiver.
- Weekly phone contact should be performed from Transplant Center office to all patients, assessing health status, symptoms and contacts at risk.

### Laboratory test screening and monitoring

Weekly NPS and monthly serology should be performed (*Figure*s *1* and *2*). Whenever possible, door‐to‐door NPS should be performed, granting greater patient's safety at home. In‐house rRT‐PCR assays are performed, targeting one or more genes in the SARS‐CoV‐2 genome, with a specificity close to 100%.^5^ The rate of rRT‐PCR detection of SARS‐CoV‐2 in patients diagnosed with COVID‐19 is 93% in bronchoalveolar lavage fluid, but then decreases to 63% in nasal swabs, and it is only 32% in pharyngeal swabs.^5^, ^6^ Of note, some of initially false‐negative test results may then turn later positive, when swabs are re‐collected some days after initial testing.^5^, ^7^

**FIGURE 1:**
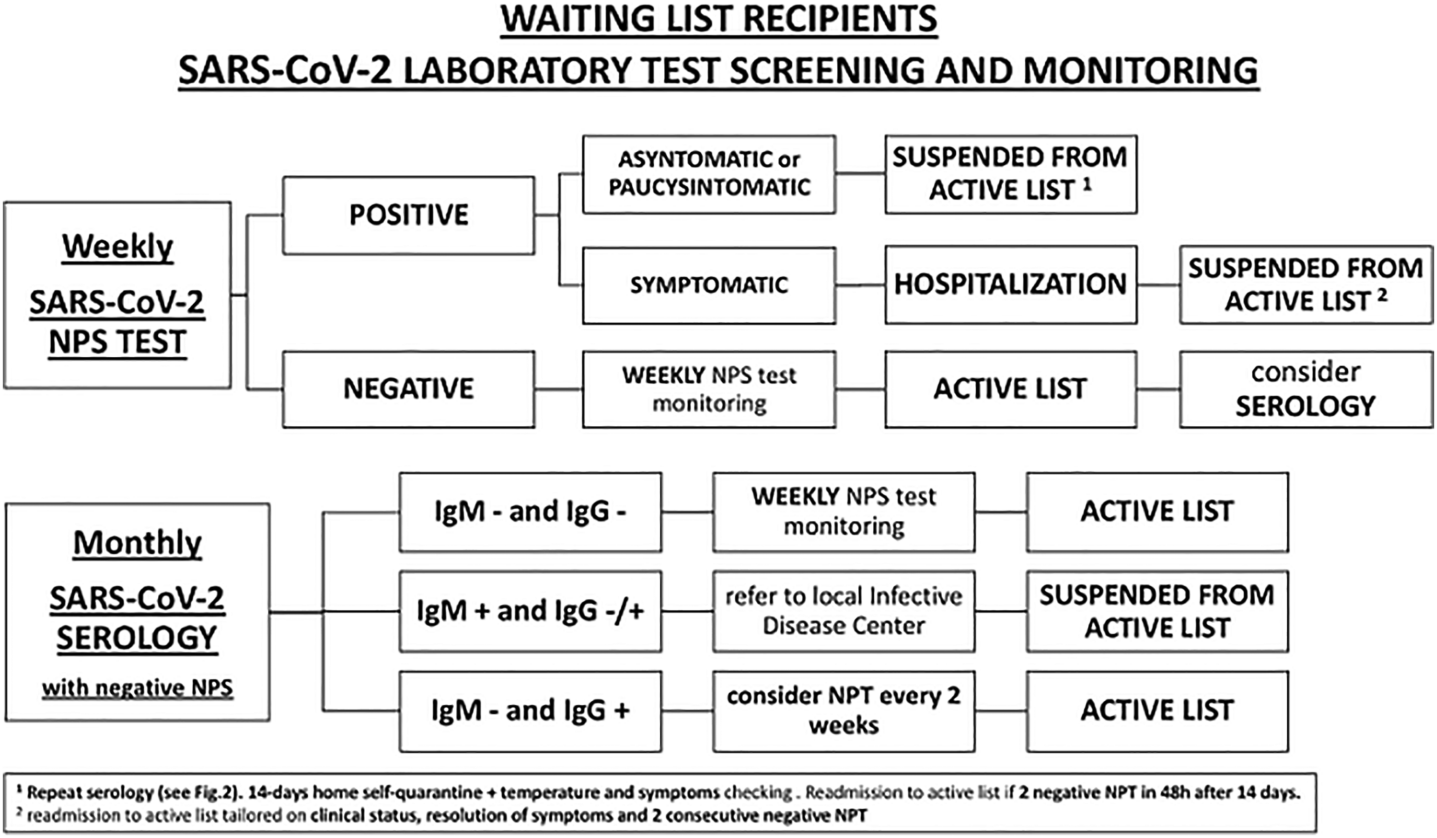
Algorithm for waiting list recipient's SARS‐CoV‐19 test screening and monitoring.

**FIGURE 2:**
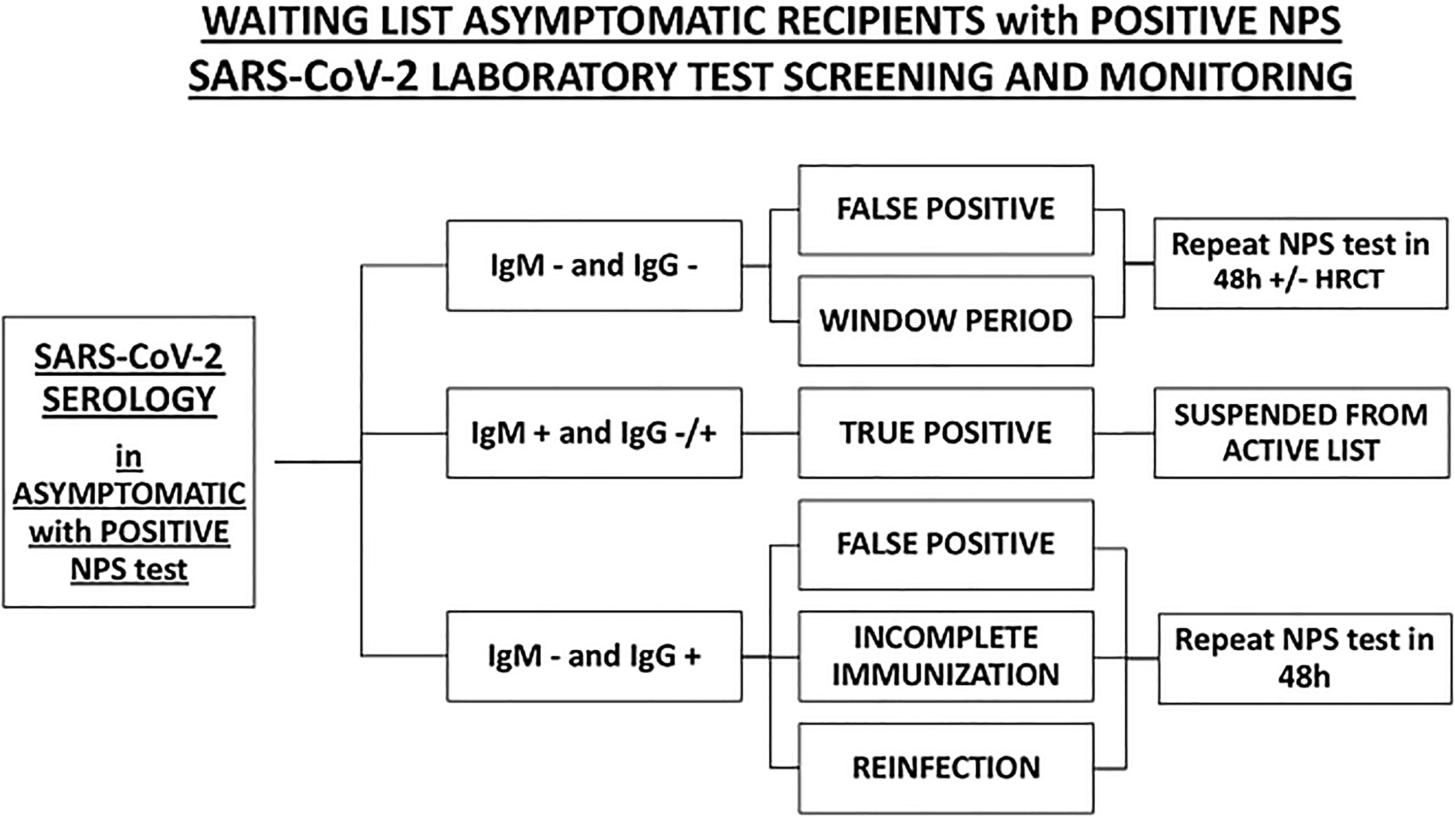
Algorithm for asymptomatic recipients with positive NPS laboratory test assessment and monitoring.

For these reasons, we decided to explore serology too, through ELISA assays, when the patient is tested for the first time, and then monthly. Serology testing became clinically available during the second half of April. This type of testing is not meant to replace NPS for etiological diagnosis of COVID‐19, but rather to have a double check and/or a deeper investigation of eventual immune response presence to SARS‐CoV‐2 exposition. Despite a specificity greater than 95%, the rate of detectable anti‐SARS‐CoV‐2 IgM could range from 50% to 75%, depending on clinical phase of infection, and it is usually superior to 90% for IgG.^5^ Moreover, CT scan should be considered when a graft is assigned, as it can detect typical ground glass and interstitial pneumonia aspects days before positivity of NPS and of symptoms occurrence.^8^

Considering later result of serology, compared to NPS, the following combinations could be present:

- NP swab and r‐RT‐PCR:
  ○ if *negative*: continue weekly test monitoring; the patient stays on active list.
  ○ if *positive*: patient is referred to the local infectious disease centre and is suspended from the active list.
    ▪ Immediate isolation of patient and caregiver.
    ▪ If asymptomatic or paucisymptomatic: activate home self‐quarantine, with daily temperature monitoring and reporting of onset of symptoms.
    ▪ Repeat serology (if not collected with the NPS at the same time):
      • if *no markers* of previous exposition to COVID‐19 (*negative IgM and IgG*) are present: false NPS positive or window period should be suspected, repeat NPS at 48 h, *consider high‐resolution CT scan (HRCT), if available*.
      • if markers of *active infection* (*positive IgM and negative/positive IgG*) are present: the infection is confirmed.
      • if markers of *past infection* (*negative IgM and positive IgG*) are present: false positive NPS or incomplete immunization or re‐infection should be suspected; repeat NPS at 48 h.
- Serology assessment (when negative NPS):
  ○ if *no markers* are present: proceed with surveillance protocol; the patient stays on active list.
  ○ if markers of *active infection* (*positive IgM and negative/positive IgG*) are detected: we should suspect NPS false negative; immediate refer to local infectious disease outpatient and follow local procedure; repeat NPs at 48 h, *consider HRCT;* the patient is suspended from the active list.
  ○ if markers of *past infection* (*negative IgM and positive IgG*) are present: test for presence of neutralizing antibodies and consider a reduction in weekly NPS monitoring; the patient stays on active list.

### When a compatible organ becomes available

- Matched recipient will be telephonically contacted and he/she has to confirm asymptomaticity, no previous risky contacts and recent (<1 week) negative NPS, normal body temperature. Patient has to be contacted before organ assignment.
- As soon as possible, ultra‐rapid NPS should be repeated and result should be known before organ retrieval. If time to NPS test result is more than organ delivery time, *consider HRCT to further support supposed infection absence*.

## Peri/postoperative protocol

### General prevention measures

- After surgery, the patient will be admitted to the intensive care unit. Ensure a single isolated room and a dedicated nurse for recipient.
- Relatives' visits are not allowed during the hospital stay. Patient's clinical information will be communicated telephonically to one designed relative.

### Laboratory test screening and monitoring

**FIGURE 3:**
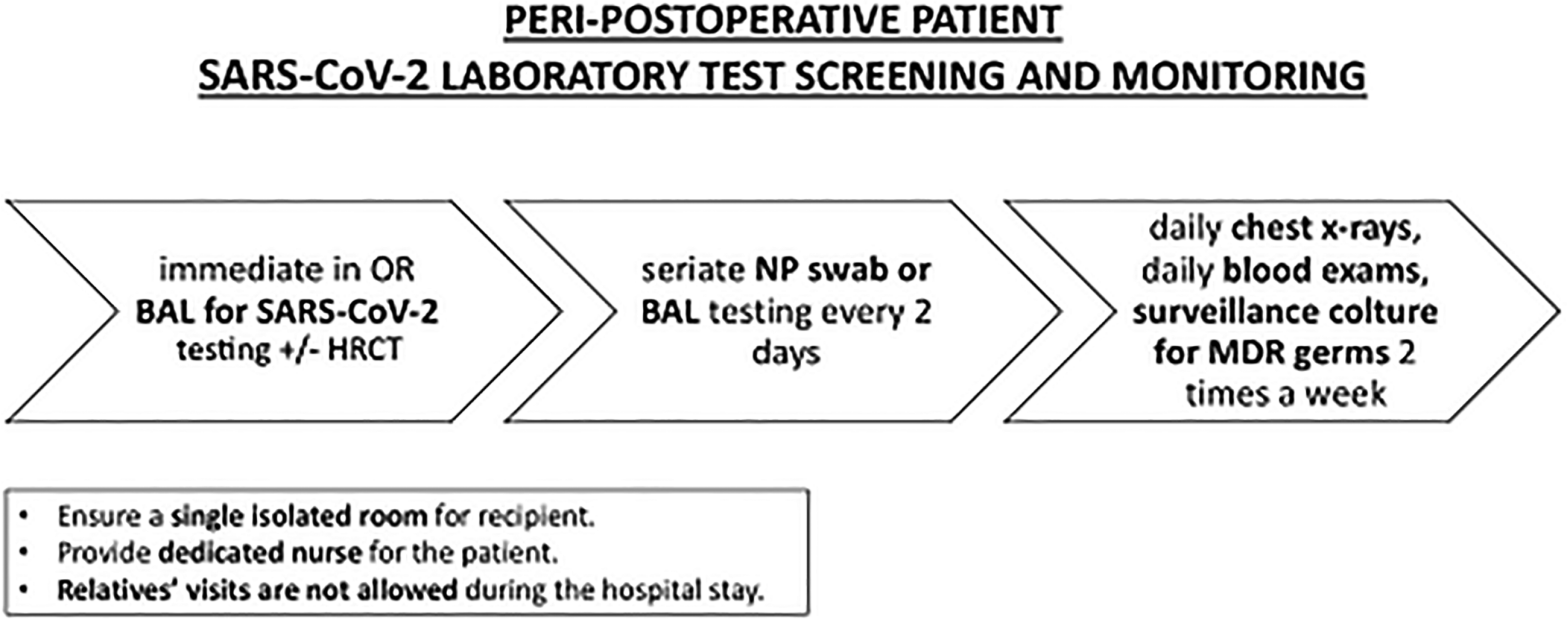
Peri‐postoperative transplanted patient laboratory test protocol.

- Immediate broncho‐alveolar lavage (BAL) fluid testing for SARS‐CoV‐2 at the time of admission in intensive care unit should be performed (*Figure* 3).
- Patient will undergo seriate NPS every other day. The incubation period is usually between 2 and 14 days in the general population,^9^ although longer incubations have been documented.^10^
- If patient is intubated, test will be performed on BAL lavage fluid preferentially.
- During intensive care unit stay, patient will undergo daily chest X‐rays, daily blood exams, and surveillance cultures for multi‐drug‐resistant bacteria twice a week (rectal swab, bronchial aspirate, and blood culture from arterial and venous access sites) or when clinically indicated.

### Antibiotic/antifungal prophylaxis management

- If the patient is clinically stable, COVID‐19 test is negative, and no suspicious signs of infection are present at blood and instrumental exams, the standard institutional antibiotic prophylaxis regimen for heart transplant recipient will be adopted (*teicoplanin*, *piperacillin‐tazobactam*, *levofloxacine*).
- In case of positive COVID‐19 test, immediate consultation with infectious disease specialist will be established after performing chest CT scan.
- Implementation of antibiotic prophylaxis with second‐line drugs or antifungal drugs will be discussed. Subsequent COVID‐19 tests will be performed according to clinical course.

### Immunosuppressive therapy management

- If patient is clinically stable, COVID‐19 tests are negative and no suspicious signs of bacterial/fungal infection are present, the standard institutional immunosuppressive therapy (anti‐thymocyte globulin for the first 3 days + steroids at decreasing dosage + cyclosporine from the third day) will be adopted. Surveillance endomyocardial biopsies (EMB) will be scheduled according to local institutional protocol.
- The impact of immunosuppression on COVID‐19 is not currently known, but decreasing immunosuppressive therapy should be considered for infected recipients, if no recent rejection.^11^

### Inotropic/mechanical support

- In case of COVID‐19 active infection and concomitant hemodynamic instability, COVID‐mediated myocarditis should always be considered and EMB should be discussed. Mechanical Circulatory Support has to be taken in consideration.

## Actual COVID‐19 prevalence data in our transplant population

Since 1985, our Institution has performed 1001 heart transplants; to date 396 patients are still alive, 6 patients have developed active SARS‐CoV‐2 infection, and 2 of them died. Our active transplant waiting list is composed by 25 recipients (seven of them are supported with ventricular assist devices). *Since the first of March 2020, we have applied our transplant list management protocol, resulting in three successful heart transplantations in COVID‐19 negative recipients. Compared to 2019, the same number of heart transplant was performed during the considered period*.

No unexpected infection was diagnosed in patients who underwent heart transplanted since the first of March.

Only one patient on active list has developed SARS‐CoV‐2 infection, confirmed by seriate NPS and radiological findings; therefore, he has been suspended from waiting list.

## Conflict of interest

None declared.

## References

[1] World Health Organization (WHO) . Coronavirus disease (COVID‐2019) situation reports. https://www.who.int/emergencies/diseases/novel‐coronavirus‐2019/situation‐reports (8 April 2020)

[2] Siddiqu HK , Mehra MR . COVID‐19 illness in native and immunosuppressed states. A clinical‐therapeutic staging proposal. Journal of Heart and Lung Transplantation 2020; 39: 405–407. 10.1016/j.healun.2020.03.012 PMC7118652 32362390

[3] Istituto Superiore di Sanità ‐ Centro Nazionale Trapianti . Prot. 503/CNT 2020 ‐ Aggiornamento delle misure di prevenzione della trasmissione dell'infezione da nuovo Coronavirus (SARS‐CoV‐2) in Italia attraverso il trapianto di organi, tessuti e cellule. http://www.trapianti.salute.gov.it/ (3 March 2020)

[4] Istituto Superiore di Sanità ‐ Centro Nazionale Trapianti . Prot. 592/CNT 2020 – Indicazioni sull'effettuazione del tampone per la ricerca di SARS‐CoV‐2 nei riceventi di trapianto d'organo da donatore vivente e donatore deceduto. http://www.trapianti.salute.gov.it/ (16 March 2020)

[5] Lippi G , Mattiuzzi C , Bovo C , Plebani M . Current laboratory diagnostics of coronavirus disease 2019 (COVID‐19). Acta Bio Med [Internet] 2020; 91: 137–145. 10.23750/abm.v91i2.9548 PMC7569648 32420937

[6] Wang W , Xu Y , Gao R , Lu R , Han K , Wu G , Tan W . Detection of SARS‐CoV‐2 in different types of clinical specimens. JAMA 2020; 323: 1843–1844. 32159775 10.1001/jama.2020.3786 PMC7066521

[7] Zou L , Ruan F , Huang M , Liang L , Huang H , Hong Z , Yu J , Kang M , Song Y , Xia J , Guo Q , Song T , He J , Yen HL , Peiris M , Wu J . SARS‐CoV‐2 viral load in upper respiratory specimens of infected patients. N Engl J Med 2020; 382: 1177–1179. 32074444 10.1056/NEJMc2001737 PMC7121626

[8] Ai T , Yang Z , Hou H , Zhan C , Chen C , Lv W , Tao Q , Sun Z , Xia L . Correlation of chest CT and RT‐PCR testing in coronavirus disease 2019 (COVID‐19) in China: a report of 1014 cases. Radiology 2020, Feb; 26: 200642. 10.1148/radiol.2020200642 PMC7233399 32101510

[9] Center for Disease Control and Prevention (CDC) . Symptoms of coronavirus. https://www.cdc.gov/coronavirus/2019‐ncov/symptoms‐testing/symptoms.html (20 March 2020)

[10] Bai Y , Yao L , Wei T , Tian F , Jin D , Chen L , Wang M . Presumed asymptomatic carrier transmission of COVID‐19. JAMA 2020. Feb 21; 323: 1406. 32083643 10.1001/jama.2020.2565 PMC7042844

[11] Aslam S , Mehra MR . COVID‐19: yet another coronavirus challenge in transplantation. J Heart Lung Transplant 2020. S1053‐2498(20)31468‐6; 39: 408–409. 32253113 10.1016/j.healun.2020.03.007 PMC7141445

